# Indian Almond (*Terminalia catappa* Linn.) Leaf Extract Extends Lifespan by Improving Lipid Metabolism and Antioxidant Activity Dependent on AMPK Signaling Pathway in *Caenorhabditis elegans* under High-Glucose-Diet Conditions

**DOI:** 10.3390/antiox13010014

**Published:** 2023-12-20

**Authors:** Yebin Kim, Seul-bi Lee, Myogyeong Cho, Soojin Choe, Miran Jang

**Affiliations:** 1 Department of Smart Food and Drug, Inje University, Gimhae 50834, Republic of Korea; dpqls1812@oasis.inje.ac.kr (Y.K.); seul2022@oasis.inje.ac.kr (S.-b.L.); whayrud1@oasis.inje.ac.kr (M.C.); 2 Department of Food Technology and Nutrition, Inje University, Gimhae 50834, Republic of Korea; tnwls4696@live.inje.ac.kr

**Keywords:** Indian almond (*Terminalia catappa* Linn.), *Caenorhabditis elegans*, high-glucose diet, obesity, antioxidant

## Abstract

This study aimed to evaluate the antioxidant and antiaging effects of Indian almond (*Terminalia catappa* Linn.) leaf extract (TCE) on high-glucose (GLU)-induced obese *Caenorhabditis elegans*. Since TCE contains high contents of flavonoids and phenolics, strong radical scavenging activity was confirmed in vitro. The stress-resistance effect of TCE was confirmed under thermal and oxidative stress conditions at nontoxic tested concentrations (6.25, 12.5, and 25 μg/mL). GLU at 2% caused lipid and reactive oxygen species (ROS) accumulation in *C. elegans*, and TCE inhibited lipid and ROS accumulation under both normal and 2% GLU conditions in a concentration-dependent manner. In addition, TCE proved to be effective in prolonging the lifespan of *C. elegans* under normal and 2% GLU conditions. The ROS reduction effect of TCE was abolished in mutants deficient in daf-16/FOXO and skn-1/Nrf-2. In addition, the lifespan-extending effect of TCE in these two mutants disappeared. The lifespan-extending effect was abolished even in atgl-1/ATGL-deficiency mutants. The TCE effect was reduced in aak-1/AMPK-deficient mutants and completely abolished under 2% GLU conditions. Therefore, the effect of prolonging lifespan by inhibiting lipid and ROS accumulation under the high GLU conditions of TCE is considered to be the result of atgl-1, daf-16, and skn-1 being downregulated by aak-1. These results suggest that the physiological potential of TCE contributes to antiaging under metabolic disorders.

## 1. Introduction

Obesity has the potential to progress into a more severe metabolic syndrome (MS), and also causes other MSs, such as arteriosclerosis, hyperglycemia, insulin resistance, and diabetes [1,2]. In particular, obesity resulting from high sugar intake is recognized as a fatal risk factor that increases mortality by developing into serious chronic diseases, such as cancer, cardiovascular disease, osteoarthritis, kidney disease, and liver disease [2,3,4].

*Caenorhabditis elegans* consumes food through the mouth. The ingested food ingredients are absorbed by the intestinal cells. Excess calorie intake induces fat accumulation in the intestine. The shape and size of lipid droplets produced can be observed under a microscope because the torso of *C. elegans* is transparent [5,6,7]. Therefore, *C. elegans* that have exposed to compounds that cause or inhibit obesity are suitable models for identifying whether a compound is involved in obesity [8,9]. Since *C. elegans* genes are highly homologous with mammalian genes for energy metabolism, studies using gene-deficient mutants provide insight into understanding the signaling pathway [10]. In particular, since *C. elegans* have short lifespans of less than four weeks, they can be beneficial when used to study lifespans related to various health conditions and nutrient intake [10].

AMP-activated protein kinase (AMPK) plays a central role in energy homeostasis by regulating the GLU and lipid metabolism [11]. Therefore, AMPK is an important factor when studying the relationship between various nutritional metabolic stresses and aging [12]. *C. elegans* is an important model system for studying AMPK action. In *C. elegans*, AMPK consists of two catalytic α subunits: aak-1 and aak-2 [13]. Excessive reactive oxygen species (ROS) are generated during metabolic stress, and AMPK mediates an antioxidant system that reduces ROS through the regulation of the transcription factor FOXO and Nrf2 [14]. In particular, *C. elegans* is employed to gain insight into the intervention of daf-16/FOXO in AMPK signaling pathways related to the antioxidant system [15,16,17]. Furthermore, skn-1/Nrf2 is a transcription regulator involved in the detoxification of ROS, which plays an important preservation role in stress resistance by downregulating a series of target genes involved in antioxidant enzyme activity [14]. Recent reports have shown that activated AMPK leads to a boost in Nrf2 [18]. Thus, daf-16 and skn-1 play key roles in improving oxidative stress and regulating downstream genes to extend the lifespan of *C. elegans* [19]. On the other hand, phosphorylated AMPK induces the nuclear localization of FOXO1, which binds to the promoter region to increase the expression of adipose triglyceride lipase (ATGL). ATGL, a key lipase, hydrolyzes triglycerides to form diglycerides and fatty acids [20]. This series of processes helps to extend the lifespan by regulating lipid metabolism in *C. elegans*.

To find novel health-functional material for improving the risks of MSs, we tested the benefits of Indian almond (*Terminalia catappa* Linn.) leaf extract (TCE). *T. catappa* is a large tropical tree that is native to Southeast Asia, Australia, and the Pacific. TCE contains gallic acid, corilagin, ellagic acid, rutin, kaempferol, chebulagic acid, punicalagin, punicalin, and quercetin [21,22]. In addition, TCE is known to have various medicinal effects, such as possessing anti-inflammatory, analgesic, antioxidant, and antibacterial properties, as well as liver protection and skin protection [23,24,25,26,27,28].

Since the antioxidant-activity-based cell-protective effect of TCE has been reported in various research, the in vivo lifespan extension and antiaging effects of TCE had been expected. Therefore, we induced metabolic stress in *C. elegans* through high-GLU feeding and investigated whether TCE exhibits positive physiological activity in metabolic disease models and ultimately has a longevity effect.

## 2. Materials and Methods

### 2.1. Sample Preparation

Indian almond (*Terminalia catappa*) leaves were collected in Myanmar in 2020 and their extract was received from the pharmaceutical research department (Yangon, Myanmar). The collected leaves were dried in the shade for more than 4 days until they were completely dry. All dry leaves were reflux-extracted for 16 h, using 70% (*v*/*v*) alcohol at 70 °C. The solvent was removed from the extract using a rotary evaporator (EYELA, Tokyo Rikakikai Co., Tokyo, Japan) at 60 °C. *Terminalia catappa* leaf extract (TCE) was dissolved in 10 mg/mL of dimethyl sulfoxide (DMSO), prepared as a stock solution, and stored at −80 °C until analyzed.

All reagents used in our study were HPLC- or molecular-biology-grade. Unless stated otherwise, all the materials were purchased from Sigma Chemical Co. (St. Louis, MO, USA).

### 2.2. LC-MS/MS Analysis

To identify phenolic compounds in TCE, we used UPLC-QTOF-MS. The analytical conditions are shown in Table 1. Solvents used for analysis were of HPLC grade and purchased from Millipore (Bedford, MA, USA).

### 2.3. Total Phenol Contents (TPC) and Total Flavonoid Contents (TFC)

The total phenolic contents (TPC) in the TCE were determined with the Folin–Ciocalteu (FC) method. Briefly, 700 μL sample (mixed with FC in 1:1 ratio) and 700 μL sodium carbonate were mixed and placed in the dark for 1 h. An absorbance reading was performed at 720 nm. The total phenolic contents of the sample were calculated using the equation for the gallic acid standard curve. The results were expressed as gallic acid equivalents (GAE)/g.

To determine the total flavonoid contents (TFC), 500 μL of the sample, 1.5 mL of sodium nitrite (5%), 10 μL of aluminum chloride (10%), and 1 mL of sodium hydroxide (1 N) were mixed. The absorbance was measured at 415 nm [29]. The total flavonoid contents of the samples were calculated using the equation for the quercetin standard curve. The results were expressed as quercetin equivalents (QE)/g.

### 2.4. Radical Scavenging Capacity

To measure the scavenging capacity against 2,2-azino-bis-3-ethylbenzothiazoline-6-sulphonic acid (ABTS) radical, we modified and used the method from Gullon et al. [30]. To produce ABTS cations, the mixture containing 7.0 mM ABTS (in 20 mM sodium acetate buffer, pH 4.5) and 2.45 mM potassium persulfate was kept in the dark overnight. The ABTS solution was diluted to an absorbance of 0.7 ± 0.01 at 734 nm. An amount of 50 µL of samples reacted with 950 µL of ABTS solution and then was placed for 10 min at room temperature. An absorbance at 734 nm was recorded.

A 2,2-diphenyl-1-picrylhydrazyl (DPPH) assay was modified by Li et al. [31]. Simply, a sample of the same volume and a DPPH solution (0.2 mM) were mixed and left at room temperature for 30 min. The reaction mixture was recorded at 517 nm using a microplate reader (microplate reader SYNERGY HTX, Biotek, CA, USA).

ABTS and DPPH radical-scavenging activity was calculated using the following formula: radical scavenging activity (%) = [1 − (sample O.D./blank O.D.)] × 100

Furthermore, we presented the value of the antioxidant capacity, which was calculated using an ascorbic acid (AA) calibration curve and expressed in micrograms of AA per gram of sample dry weight (DW).

### 2.5. Worm Study

#### 2.5.1. Cultivation

For experimental use of *C. elegans* strain N2 (wild-type) and its derivative mutant strains, skn-1 (zu67) IV and daf-16::GFP (zls356) were obtained from the Caenorhabditis Genetics Center (CGC) in the U.S.A. Strains daf-16 (tm5030), atgl-1 (tm12352), and aak-1 (tm1944) were obtained from the National BioResource Project (NBRP) of Japan.

All strains were kept on nematode growth medium (NGM) plates that were spread with *E. coli* OP50. They were maintained at a temperature of 20 °C throughout the entire experiment. Age synchronization of nematodes was achieved by separating the eggs from gravid adults using a solution containing 6% sodium hypochlorite and 5 M NaOH [32].

#### 2.5.2. Acute Toxicity

Synchronized L4 was washed twice with M9 buffer and suspended in M9 buffer containing cholesterol. One milliliter of the suspension was transferred to each well of a 24-well plate (20–30 worms/well) and mixed with 10 μL of various concentrations of TCE. Then, the plates were left at 20 °C for 24 h. Acute toxicity results were expressed as percent survival after counting the live worms.

#### 2.5.3. Oil Red O Staining

To determine lipid contents, oil red O staining was partially modified and performed [33]. Simply, cultivated worms were fixed in 4% formaldehyde for 24 h, then dehydrated with 60% isopropanol at −70 °C for 15 min. The dehydrated worms were washed three times with M9 buffer and dyed with an oil red O solution for 2 h. The stained worms were washed with M9 buffer, then observed under a microscope (Nikon, Seoul, Republic of Korea). The relative strength of the stained lipid droplets in the worms was quantified using Image J 1.8.0. software.

#### 2.5.4. Triglyceride (TG) Assay

The cultured L4 worms were resuspended in 500 μL PBS containing 0.05% Tween-20 solution. They were homogenized on ice using a glass homogenizer (ALLSHENG, regional, national) for 5 min to collect the pellet and then centrifuged at 1000× *g* for 5 min. The obtained supernatant was analyzed for TG content. TG content was measured using the absorbance at 570 nm with a TG kit (Biomax, Guri, Republic of Korea).

#### 2.5.5. Determination of Stress Resistance

In the case of thermal and oxidative stress analysis, 50 synchronized N2 L1 larvae were adjusted as follows. OP50 and TCE were mixed and seeded on NGM plates.

To evaluate the effect on thermal stress, the worm plate was maintained at 20 °C for 72 h, incubated at 35 °C for 4 h, and then transferred back to 20 °C. In addition, to test the effect on oxidative stress, pretreated worms were then transferred to a 24-well plate containing a concentration of 80 μM juglone (5-hydroxy-1,4-naphthoquinone, Sigma-Aldrich, St. Louis, MI, USA). The plate was incubated at 20 °C.

After incubation, the living and the dead worms were counted and subsequently scored, as described in the study conducted by Rathor et al. [34].

#### 2.5.6. DCF–DA Assay

L1 worms were treated with different concentrations of extracts for 64 h and then incubated with 100 μM H_2_DCF-DA for 1 h in the dark. After that, the nematodes were mounted in NAN_3_ (2%) onto microscope slides. The slides were viewed using a Nikon ECLIPS Ci (Nikon, Seoul, Republic of Korea) fluorescence microscope. The fluorescence intensities were examined using Image J software. An average of 15 worms per group was selected for quantification.

#### 2.5.7. Daf-16 Nuclear Localization

Daf-16 (zIs356) nematode is daf-16::GFP mutant, which is a transgenic mutant nematode fused with green fluorescent protein and daf-16. After daf-16 mutants in the L1 stage were treated with extracts for 64 h, they were immobilized on microscope slides with 2% NaN_3_. The patterns of daf-16::GFP expression were evaluated as “cytosolic”, “intermediate”, and “nuclear”, which counted the percentage of each treatment group.

#### 2.5.8. Lifespan Assay

Young adult nematodes were transferred to M9 buffer with cholesterol. Then, different concentrations of extracts were added, including 120 μM of 5-Fluoro-2’-deoxyuridine (FUDR, 98%) (Alfa Aesar, Seoul, Republic of Korea), to prevent spawning, and carbenicillin (50 μg/mL), to prevent bacterial infection. The transfer day was designated as day 1, the old medium was removed and fresh medium containing the extract was added every other day until all worms died. The results were expressed as a percentage of the survival rate (% survival rate).

#### 2.5.9. Statistical Analysis

Results are presented as the means ± standard deviations (SD) of three independent replicates. The significance of intergroup differences was determined using one-way analysis of variance (ANOVA), followed by Tukey’s multiple range test. SPSS 27.0 was used for all statistical analyses except lifespan. *p*-values < 0.05 were considered to be significant. The results of the lifespan and the mean lifespan were analyzed with the Kaplan–Meier method using the OASIS application (https://sbi.postech.ac.kr/oasis/, accessed on 21 July 2023). *p*-values of survival differences were determined with the log-rank test.

## 3. Results

### 3.1. TCE Components Profile

**Figure 1:**
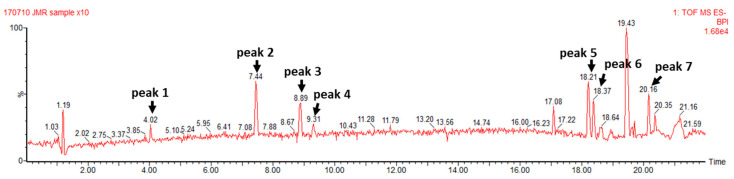
UPLC-QTOF-MS chromatogram of TCE. Peak 1: gallic acid, peak 2: orientin, peak 3: vitexin, peak 4: ellagic acid, peak 5: arjungenin, peak 6: arjunolic acid, and peak 7: betulinic acid.

As a result of the UPLC-QTOF-MS analysis, seven compounds were identified as bio-phenolic components of TCE (Figure 1), including phenolic acids such as gallic acid and ellagic acid, flavonoids such as orientin, vitexin, and terpenes or terpenoids such as arjungenin, arjunolic acid, and betulinic acid. The molecular weight of each peak is presented in Figure S1.

### 3.2. Total Phenol and Total Flavonoid Contents and Antioxidant Activity of TCE

The total phenol content (TPC) and the total flavonoid content (TFC) of TCE were 693.65 ± 60.3 GAE/g and 268.39 ± 10 QE/g, respectively. The results were expressed as 50% inhibition concentration (IC50) and trolox equivalents (TE) (Table 2). The DPPH scavenging capacity and IC50 of TCE were 84.33 ± 0.23 μTE/g and 18.82 ± 0.04 μg/mL, respectively. The ABTS scavenging capacity and IC50 of TCE were 2.23 ± 0.09 μTE/g and 125.77 ± 0.47 μg/mL, respectively (Table 2).

### 3.3. Safety of TCE

**Figure 2:**
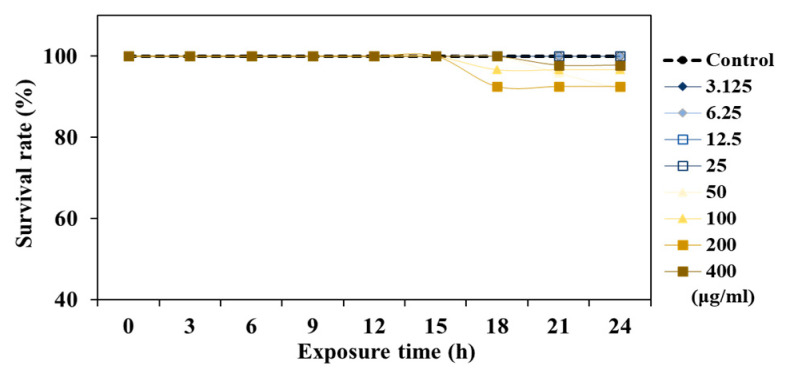
Safety of TCE at different concentrations (3.125–400 μg/mL).

To evaluate the safety of TCE, acute toxicity tests were performed with different doses of TCE (3.125–400 μg/mL). No toxicity was observed at 3.125–25 μg/mL, but worm survival was reduced in the concentration range of 50–400 μg/mL of TCE, which was determined to be a toxic concentration. Therefore, safety concentrations (6.25 μg/mL, 12.5 μg/mL, 25 μg/mL) of TCE were treated in further experiments (Figure 2).

### 3.4. Inhibitory Effect on Lipid Accumulation of TCE

**Figure 3:**
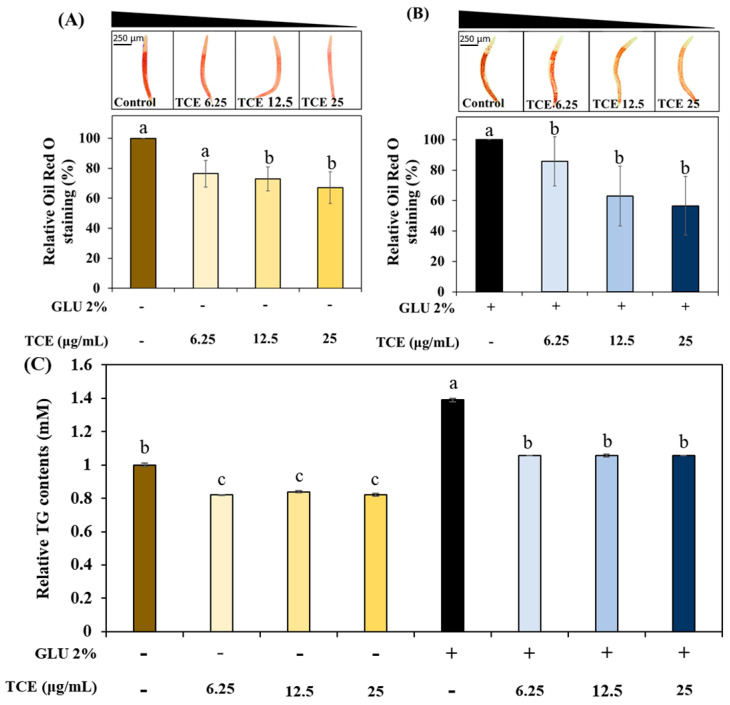
Inhibition of TCE on lipid accumulation. Total fat contents under (**A**) normal and (**B**) 2% GLU (glucose) conditions. (**C**) TG content in the worms (more than 2000 worms were used in each independent experiment). Bars represent the means ± SDs (*n* = 3 plates and 10 worms for oil red O staining assay). One thousand worms were used in each independent group for the TG assay. Statistical analysis was performed using one-way ANOVA, followed by Tukey’s post hoc test. Different letters above bars mean statistically significant differences (*p* < 0.05).

To understand the work of TCE on lipid metabolism, we investigated the effect of TCE on fat accumulation in *C. elegans* under normal or 2% GLU conditions. The TCE group exhibited a concentration-dependent decrease in total fat accumulation in normal and 2% GLU conditions compared to the control group (Figure 3A,B). Consistently, the TCE-treated worms had reduced triglyceride (TG) content compared to the control group under both normal and 2% GLU-treated groups (Figure 3C).

### 3.5. Evaluation of TCE Stress Resistance under Various Stress Conditions

**Figure 4:**
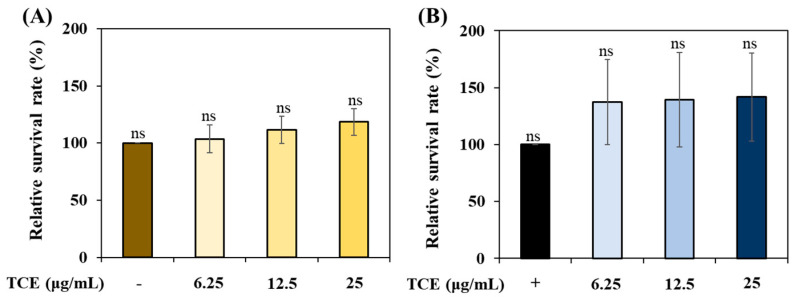
The stress resistance of TCE under (**A**) oxidative stress condition and (**B**) thermal stress condition. Bars represent the means ± SDs (*n* = 3 plates and 10 worms). Statistical analysis was performed using one-way ANOVA, followed by Tukey’s post hoc test (*p* < 0.05). ns: no significance.

To evaluate the stress resistance of TCE, we analyzed its ability to improve resistance to thermal and oxidative stress. TCE showed an increase in survival compared to the control group under thermal stress (Figure 4A) and oxidative stress (Figure 4B) conditions. However, there was no statistically significant difference.

### 3.6. Inhibitory Effects on Glucose-Induced ROS Accumulation of TCE

**Figure 5:**
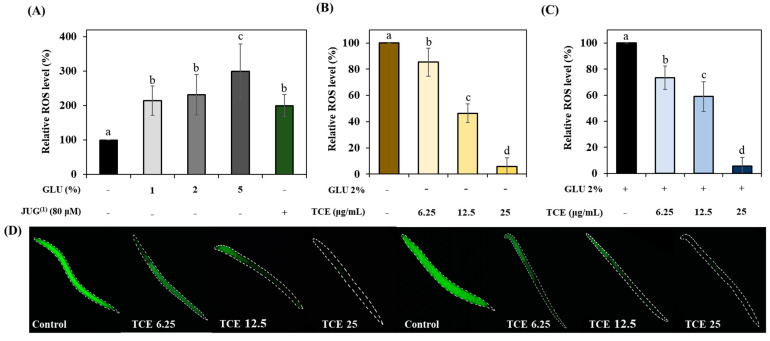
The inhibitory effect on ROS accumulation of TCE. (**A**) GLU (glucose)-induced oxidative stress and ROS content under (**B**) normal and (**C**) 2% GLU conditions. (**D**) Images of green-fluorescent-stained ROS under normal and 2% GLU conditions. Bars represent the means ± SDs (*n* = 3 plates and 10 worms). Statistical analysis was performed using one-way ANOVA, followed by Tukey’s post hoc test. Different lowercase letters means statistically significant differences (*p* < 0.05). (1) Juglone.

We investigated whether GLU can induce oxidative stress. Treatment of GLU at concentrations of 1%, 2%, and 5% revealed that GLU induces oxidative stress in a concentration-dependent manner, which is similar to 80 μM juglone (Figure 5A). TCE-treated N2 worms resulted in a reduction in oxidative stress compared to the control group under normal (Figure 5B,D) and even GLU (Figure 5B,D) conditions.

### 3.7. TCE Effect on Lifespan Prolonging

**Figure 6:**
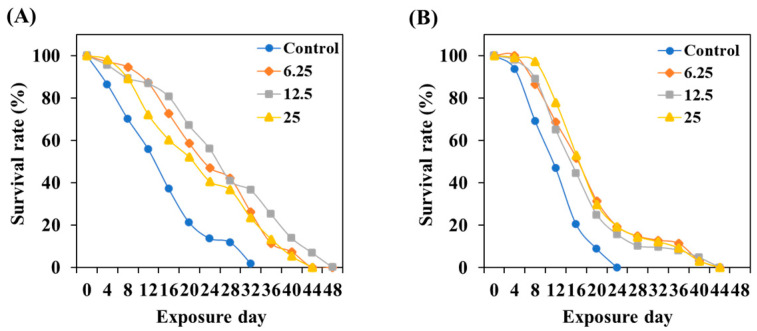
Effect of TCE on lifespan in wild-type worms under (**A**) normal and (**B**) 2% GLU (AD) conditions. Fifty worms were used in each independent group.

To evaluate the effect of TCE on the lifespan of *C. elegans*, tested worms were incubated until all worms died while preventing progeny production at 20 °C. The results indicated that TCE treatment can extend the lifespan of cultured *C. elegans* under normal conditions by a maximum of 16 days (Figure 6A). Even in GLU conditions, the TCE treatment extended the lifespan of *C. elegans* by a maximum of 20 days (Figure 6B).

### 3.8. Role of Daf-16 in TCE Effect

**Figure 7:**
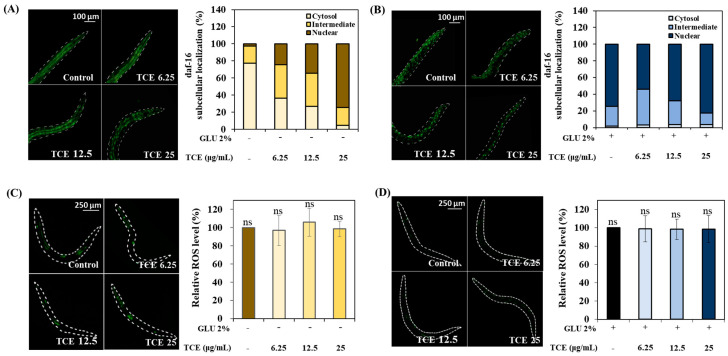
*Daf-16* nuclear localization and determination of ROS level in *daf-16* null mutants. *Daf-16* nuclear localization under (**A**) normal and (**B**) 2% GLU (glucose) conditions. ROS levels under (**C**) normal and (**D**) 2% GLU (glucose) conditions. Bars represent the means ± SDs (*n* = 3 plates and 10 worms). Statistical analysis was performed using one-way ANOVA, followed by Tukey’s post hoc test (*p* < 0.05). ns: no significance.

To investigate localization to the nucleus of *daf-16* due to the TCE treatment, L1 larvae of *daf-16*::GFP were treated with TCE for 64 h. In TCE-treated worms, the intensity of the *daf-16* nuclear replacement increased compared to the control group in a concentration-dependent manner (Figure 7A). Similarly, it was observed that the nuclear expression of *daf-16* increased compared to the control group in TCE-treated worms that were also in the GLU condition (Figure 7B).

To precisely identify the role of *daf-16* in TCE action, the ROS intensity was analyzed using *daf-16* null worms. *Daf-16* null worms were treated with TCE; there was no significant difference observed in ROS expression under both normal and GLU conditions (Figure 7C,D). As a result, the ROS-reducing effect of TCE in the wild type was abolished, suggesting that *daf-16* was involved in the TCE effect.

### 3.9. Role of Skn-1 in TCE Effect

Besides identifying the relationship between the action of TCE and the role of *daf-16*, we also investigated the impact of TCE on *skn-1* transcription factors by utilizing *skn-1* knockdown worms.

**Figure 8:**
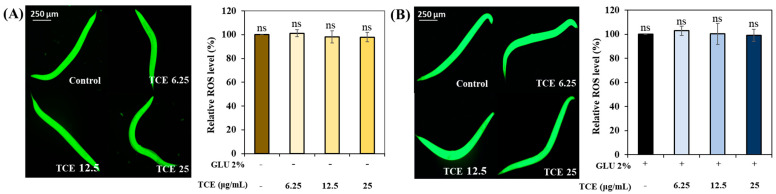
Determination of ROS level in *skn-1* knockdown mutants. (**A**) ROS level under normal condition. (**B**) ROS level under GLU condition. Bars represent the means ± SDs (*n* = 3 plates and 10 worms). Statistical analysis was performed using one-way ANOVA, followed by Tukey’s post hoc test (*p* < 0.05). ns: no significance.

Similar to the daf-16 result, there was no significant difference in the ROS expression between the control and the TCE-treatment group in both normal and GLU conditions in skn-1 null worms (Figure 8A,B). These results suggest that the expression of *skn-1*, along with *daf-16*, affects the TCE effect.

### 3.10. The Role of Various Metabolic Stress Regulators in the Lifespan-Extending Effect of TCE

To gain insight into how TCE interacts with the various genes that regulate energy metabolism, we used *daf-16*, *skn-1*, *atgl-1*, and *aak-1* knockdown mutant worms to evaluate the effect of TCE on longevity.

*Daf-16*, *skn-1*, *atgl-1*, and *aak-1* gene knockdown mutants were treated with different concentrations of TCE, respectively. In earlier experiments, the lifespan of the wild-type, N2 was extended depending on the concentration of the treated TCE (Figure 5). However, the lifespan of the four mutants ended similarly, with no statistical difference according to the TCE concentration (Figure 8, Table S1). This result means that the lifespan-extending effect of TCE is related to the *daf-16*, *skn-1*, *atgl-1*, and *aak-1* genetic factors.

## 4. Discussion

Recent research suggests that obesity is not simply attributable to caloric imbalance but is influenced by systemic factors associated with insulin metabolism [35,36,37]. Therefore, we induced metabolic disorders using an excessive GLU intake model and evaluated the level of accumulated fat, a representative phenotype of obesity. Our results showed that the treatment of TCE in high-GLU-ingested worms reduces fat and TG accumulation (Figure 3). These results suggest that TCE can prevent high-sugar-induced obesity by primarily suppressing body fat accumulation.

Excessive secretion of insulin due to a high-sugar diet can trigger systemic oxidative stress, inflammation, and aging. These metabolic stresses contribute to the pathogenesis of obesity and diabetes, which can subsequently lead to various complications [38,39,40]. Our study found that 2% GLU caused ROS accumulation of as much as 80 μM JUG. Numerous studies have verified hypotheses suggesting a link between GLU-diet-derived ROS overproduction and lifespan, and they support our results [41,42]. However, TCE exhibited a protective effect against GLU-induced oxidative damage (Figure 5).

**Figure 9:**
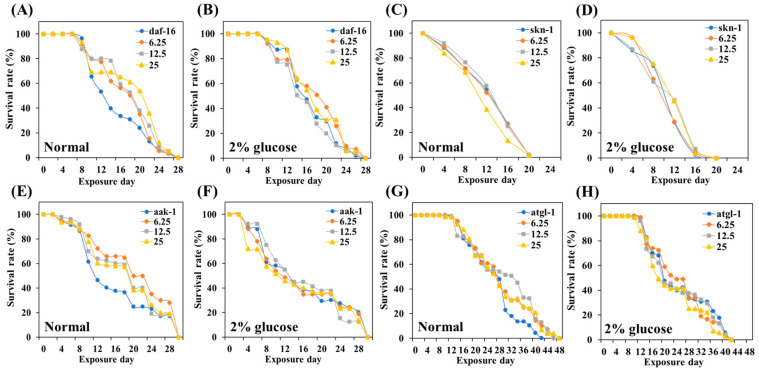
Effect of TCE on lifespan in various mutants. (**A**,**B**) survival curve of daf-16 under normal and GLU conditions. (**C**,**D**) Survival curve of skn-1 under normal and GLU conditions. (**E**,**F**) Survival curve of aak-1 under normal and GLU conditions. (**G**,**H**) Survival curve of atgl-1 under normal and GLU conditions. Fifty worms were used in each independent group.

Normally, transcription factors daf-16/FOXO and skn-1/Nrf2 are located primarily in the cytoplasm, but when exposed to internal or external stress, these factors translocate into the nucleus [43,44]. Nuclear expression of these factors initiates a series of stress controls, such as regulating the activity of antioxidant enzymes to prevent ROS overproduction [45]. The inhibitory effect of TCE, as well as the promotional effect of GLU on ROS production in *daf-16* and *skn-1* knockdown mutations, has been abolished (Figure 8). Furthermore, the lifespan-extending effect of TCE has been abolished in daf-16 and skn-1 mutants (Figure 9). Therefore, we clearly confirmed that the in vivo antioxidant effect of TCE on GLU-induced oxidative toxicity is related to daf-16 and skn-1, and we conclude that the longevity effect of TCE is based on the antioxidant defense system. Daf-16/FOXO and skn-1/Nrf2 are downstream transcription factors expressed by the activation of aak-1/AMPK [46,47]. In our lifespan result of the aak-1 knockdown mutant, The TCE effect shown in N2 was abolished (Figure 9). Taken together, it is believed that the inhibitory effect of TCE against oxidative stress ultimately contributed to extending the lifespan of *C. elegans* (Figure 6). The in vitro antioxidant effect of TCE has been verified through numerous studies, and the preventive effects on skin aging and osteoporosis related to antioxidant properties have been reported [28,48]. However, the effect of TCE on lifespan extension has not been reported. In particular, this is the first discovery that the antioxidant-based metabolic disease improvement and longevity effect of TCE depends on the AMPK pathway.

Meanwhile, AMPK is a key regulator in energy metabolism and is particularly well known to control GLU and lipid metabolism [11,48]. In metabolic regulation, because AMPK activation induces health-beneficial physiological effects, AMPK is considered an important therapeutic target for controlling human diseases, including MS [49]. We found that the accumulated fat was reduced due to GLU feeding in TCE-treated worms. Thus, further investigation of the genetic factors related to lipid metabolism controlled by AMPK was needed. Fat accumulation is controlled by the balance between fat synthesis (lipogenesis) and fat degradation (lipolysis and beta-oxidation) [49]. Daf-16 is a factor that mediates the antioxidant system to respond to stress and is also involved in lipogenesis-related factors in lipid accumulation [50]. Therefore, increased daf-16 expression through TCE treatment also means that TCE intervenes in daf-16 expression to regulate fat accumulation. In addition, AMPK downregulates atgl-1/ATGL, which is a major lipase in lipolysis [51]. The TCE effect on the lifespan extension was abolished in the atgl-1 knockdown mutant, and this result implies that the effect of TCE is due to the action of atgl-1. A number of studies focusing on lipid metabolism related to AMPK activation have demonstrated that the intervention of daf-16 and atgl-1 attenuates lipid levels and prolongs their lifespan [20,46,51].

In conclusion, TCE prolonged the lifespan by improving antioxidant activity and lipid metabolism in nematode models of high-GLU-diet-induced metabolic disorders. It was confirmed that the TCE effect is influenced by aak-1/AMPK, which is responsible for energy metabolism. The activation of aak-1/AMPK regulates oxidative stress by inducing the nuclear transition of skn-1 and daf-16. In addition, AMPK downregulates daf-16 and atgl-1, which are in charge of lipogenesis and lipolysis, respectively. TCE intervened in the action of those genetic factors related to energy metabolism. In other words, it is believed that the effect of TCE depends on skn-1, daf-16, and atgl-1 belonging to the aak-1/AMPK pathway. Therefore, it has been proven that Indian almond leaves have the potential to prevent and improve metabolic diseases by regulating genetic factors through the AMPK pathway. This study is the first to experimentally demonstrate that TCE has a longevity effect by modulating AMPK to relieve metabolic stress.

## Figures and Tables

**Table 1:** UPLC-QTOF-MS analysis condition of phenolic compounds in TCE.

LC Condition (Waters^®^ ACQUITY^TM^ UPLC)	
Column	CORTECS^TM^ UPLC^®^ C18 1.6 μm (2.1 × 100 mm)
	Temperature: 35 °C
Mobile phase	(A) 0.1% formic acid in water and (B) 0.1% formic acid in MethanolGradient: 5% solvent B for 1 min, 5–30% solvent B for the next 4 min, 30–32% solvent B for 2 min, 32–50% solvent B for 7 min, 50–90% solvent B for 4 min, 90% solvent B for 2 min, and a linear step of 90-2% solvent B for 3 min.
Flow rate	0.25 mL/min
MS Condition (SYNAPT^TM^ G2)	
Ionization Mode	ESI-
Temperature	Source: 120 °C, and desolvation: 350 °C
Voltage	Capillary: 2.5 kV, sampling cone: 35 V, extraction cone: 4.0 V
Gas flow	Cone gas: 100 L/h, and desolvation gas: 800 L/h

**Table 2:** Total phenolic content (TPC), total flavonoid content (TFC), and the antioxidant activity evaluated using the DPPH and ABTS assays of TCE.

TPC ^(1)^	TFC ^(2)^	DPPH		ABTS	
		IC50 ^(3)^ (μg/mL)	μM TE ^(4)^/g	IC50 (μg/mL)	μM TE/g
0.694 ± 0.06	0.268 ± 0.011	18.816 ± 0.038	84.326 ± 0.228	125.773 ± 0.465	2.227 ± 0.09

^(1)^ Total Phenolic Contents, ^(2)^ Total Flavonoid Contents, ^(3)^ IC50: 50% Inhibition Concentration, and ^(4)^ TE: Trolox Equivalent. All values represent the means ± SDs of three separate experiments.

## Data Availability

The data presented in this study are available on request from the corresponding author.

## Supplementary Materials

The following supporting information can be downloaded at: https://www.mdpi.com/article/10.3390/antiox13010014/s1, Figure S1: Mass spectra corresponding to individual peaks obtained from UPLC-QTOF-MS. Table S1: Influence of TCE on lifespan under normal and 2% GLU (glucose) conditions in various knockdown mutants.

## References

[1] Pi-Sunyer X. (2009). The medical risks of obesity. Postgrad. Med..

[2] Francisqueti F.V., Nascimento A.F., Minatel I.O., Dias M.C., Luvizotto R.d.A.M., Berchieri-Ronchi C., Ferreira A.L.A., Corrêa C.R. (2017). Metabolic syndrome and inflammation in adipose tissue occur at different times in animals submitted to a high-sugar/fat diet. J. Nutr. Sci..

[3] Oliveira D.T.d., Fernandes I.d.C., Sousa G.G.d., Santos T.A.P.d., Paiva N.C.N.d., Carneiro C.M., Evangelista E.A., Barboza N.R., Guerra-Sá R. (2020). High-sugar diet leads to obesity and metabolic diseases in ad libitum-fed rats irrespective of caloric intake. Arch. Endocrinol. Metab..

[4] Johnson R.J., Segal M.S., Sautin Y., Nakagawa T., Feig D.I., Kang D.-H., Gersch M.S., Benner S., Sánchez-Lozada L.G. (2007). Potential role of sugar (fructose) in the epidemic of hypertension, obesity and the metabolic syndrome, diabetes, kidney disease, and cardiovascular disease. Am. J. Clin. Nutr..

[5] Wormbook. http://www.wormbook.org/chapters/www_feeding/feeding.html.

[6] McKay R.M., McKay J.P., Avery L., Graff J.M. (2003). *C. elegans*: A model for exploring the genetics of fat storage. Dev. Cell.

[7] Corsi A.K., Wightman B., Chalfie M. (2015). A transparent window into biology: A primer on *Caenorhabditis elegans*. Genetics.

[8] Dwyer D.S., Donohoe D., Lu X.H., Aamodt E.J. (2005). Mechanistic connections between glucose/lipid disturbances and weight gain induced by antipsychotic drugs. Int. Rev. Neurobiol..

[9] Zheng J., Enright F., Keenan M., Finley J., Zhou J., Ye J., Greenway F., Senevirathne R.N., Gissendanner C.R., Manaois R. (2010). Resistant starch, fermented resistant starch, and short-chain fatty acids reduce intestinal fat deposition in *Caenorhabditis elegans*. J. Agric. Food Chem..

[10] Murphy C.T., McCarroll S.A., Bargmann C.I., Fraser A., Kamath R.S., Ahringer J., Li H., Kenyon C. (2003). Genes that act downstream of DAF-16 to influence the lifespan of *Caenorhabditis elegans*. Nature.

[11] Bai J., Zhu Y., He L., Zhang J., Li J., Pan R., Zhang J., Zhao Y., Cui L., Lu H. (2022). Saponins from bitter melon reduce lipid accumulation via induction of autophagy in *C. elegans* and HepG2 cell line. Curr. Res. Food Sci..

[12] Salminen A., Kaarniranta K. (2012). AMP-activated protein kinase (AMPK) controls the aging process via an integrated signaling network. Ageing Res. Rev..

[13] Ahmadi M., Roy R. (2016). 5′-AMP-Activated Protein Kinase Signaling in *Caenorhabditis elegans*. AMP-Act. Protein Kinase.

[14] Greer E.L., Dowlatshahi D., Banko M.R., Villen J., Hoang K., Blanchard D., Gygi S.P., Brunet A. (2007). An AMPK-FOXO pathway mediates longevity induced by a novel method of dietary restriction in *C. elegans*. Curr. Biol..

[15] Ankeny R.A. (2001). The natural history of *Caenorhabditis elegans* research. Nat. Rev. Genet..

[16] Fatt H.V., Dougherty E.C. (1963). Genetic control of differential heat tolerance in two strains of the nematode *Caenorhabditis elegans*. Science.

[17] Nigon V., Dougherty E.C. (1949). Reproductive patterns and attempts at reciprocal crossing of Rhabditis elegans Maupas, 1900, and Rhabditis briggsae Dougherty and Nigon, 1949 (Nematoda: Rhabditidae). J. Exp. Zool..

[18] Kosztelnik M., Kurucz A., Papp D., Jones E., Sigmond T., Barna J., Traka M.H., Lorincz T., Szarka A., Banhegyi G. (2019). Suppression of AMPK/aak-2 by NRF2/SKN-1 down-regulates autophagy during prolonged oxidative stress. FASEB J..

[19] Zhang H., Davies K.J., Forman H.J. (2015). Oxidative stress response and Nrf2 signaling in aging. Free Radic. Biol. Med..

[20] Chen W.-L., Chen Y.-L., Chiang Y.-M., Wang S.-G., Lee H.-M. (2012). Fenofibrate lowers lipid accumulation in myotubes by modulating the PPARα/AMPK/FoxO1/ATGL pathway. Biochem. Pharmacol..

[21] Anand A., Divya N., Kotti P. (2015). An updated review of *Terminalia catappa*. Pharmacogn. Rev..

[22] Abiodun O.O., Rodríguez-Nogales A., Algieri F., Gomez-Caravaca A.M., Segura-Carretero A., Utrilla M.P., Rodriguez-Cabezas M.E., Galvez J. (2016). Antiinflammatory and immunomodulatory activity of an ethanolic extract from the stem bark of *Terminalia catappa* L. (Combretaceae): In vitro and in vivo evidences. J. Ethnopharmacol..

[23] Fan Y., Xu L., Gao J., Wang Y., Tang X., Zhao X., Zhang Z. (2004). Phytochemical and antiinflammatory studies on *Terminalia catappa*. Fitoterapia.

[24] Ratnasooriya W., Dharmasiri M., Rajapakse R., De Silva M., Jayawardena S., Fernando P., De Silva W., Nawela A., Warusawithana R., Jayakody J. (2002). Tender leaf extract of Terminalia catappa antinociceptive activity in rats. Pharm. Biol..

[25] Liu T.-Y., Ho L.-K., Tsai Y.-C., Chiang S.-H., Chao T.-W., Li J.-H., Chi C.-W. (1996). Modification of mitomycin C-induced clastogenicity by *Terminalia catappa* L. in vitro and in vivo. Cancer Lett..

[26] Nair R., Chanda S. (2008). Antimicrobial activity of *Terminalia catappa*, Manilkara zapota and Piper betel leaf extract. Indian J. Pharm. Sci..

[27] Yang S.-F., Chen M.-K., Hsieh Y.-S., Yang J.-S., Zavras A.-I., Hsieh Y.-H., Su S.-C., Kao T.-Y., Chen P.-N., Chu S.-C. (2010). Antimetastatic effects of *Terminalia catappa* L. on oral cancer via a down-regulation of metastasis-associated proteases. Food Chem. Toxicol..

[28] Wen K.-C., Shih I., Hu J.-C., Liao S.-T., Su T.-W., Chiang H.-M. (2010). Inhibitory effects of *Terminalia catappa* on UVB-induced photodamage in fibroblast cell line. Evid.-Based Complement. Altern. Med..

[29] Jang M., Choi H.Y., Kim G.H. (2019). Phenolic components rich ethyl acetate fraction of *Orostachys japonicus* inhibits lipid accumulation by regulating reactive oxygen species generation in adipogenesis. J. Food Biochem..

[30] Gullon B., Pintado M.E., Fernández-López J., Pérez-Álvarez J.A., Viuda-Martos M. (2015). In vitro gastrointestinal digestion of pomegranate peel (*Punica granatum*) flour obtained from co-products: Changes in the antioxidant potential and bioactive compounds stability. J. Funct. Foods.

[31] Li Z., Lan Y., Miao J., Chen X., Chen B., Liu G., Wu X., Zhu X., Cao Y. (2021). Phytochemicals, antioxidant capacity and cytoprotective effects of jackfruit (*Artocarpus heterophyllus* Lam.) axis extracts on HepG2 cells. Food Biosci..

[32] Pandey S., Tiwari S., Kumar A., Niranjan A., Chand J., Lehri A., Chauhan P.S. (2018). Antioxidant and anti-aging potential of Juniper berry (*Juniperus communis* L.) essential oil in *Caenorhabditis elegans* model system. Ind. Crops Prod..

[33] Bai J., Farias-Pereira R., Jang M., Zhang Y., Lee S.M., Kim Y.-S., Park Y., Ahn J.B., Kim G.-H., Kim K.-H. (2021). Azelaic acid promotes *caenorhabditis elegans* longevity at low temperature via an increase in fatty acid desaturation. Pharm. Res..

[34] Rathor L., Pant A., Nagar A., Tandon S., Trivedi S., Pandey R. (2017). *Trachyspermum ammi* L. (Carom) oil induces alterations in SOD-3, GST-4 expression and prolongs lifespan in *Caenorhabditis elegans*. Proc. Natl. Acad. Sci. India Sect. B Biol. Sci..

[35] Steinberg H.O., Chaker H., Leaming R., Johnson A., Brechtel G., Baron A.D. (1996). Obesity/insulin resistance is associated with endothelial dysfunction. Implications for the syndrome of insulin resistance. J. Clin. Investig..

[36] Wu H., Ballantyne C.M. (2020). Metabolic inflammation and insulin resistance in obesity. Circ. Res..

[37] AsghariHanjani N., Vafa M. (2019). The role of IGF-1 in obesity, cardiovascular disease, and cancer. Med. J. Islam. Repub. Iran.

[38] Fernández-Sánchez A., Madrigal-Santillán E., Bautista M., Esquivel-Soto J., Morales-González Á., Esquivel-Chirino C., Durante-Montiel I., Sánchez-Rivera G., Valadez-Vega C., Morales-González J.A. (2011). Inflammation, oxidative stress, and obesity. Int. J. Mol. Sci..

[39] Monteiro R., Azevedo I. (2010). Chronic inflammation in obesity and the metabolic syndrome. Mediat. Inflamm..

[40] Koliaki C., Liatis S., Kokkinos A. (2019). Obesity and cardiovascular disease: Revisiting an old relationship. Metabolism.

[41] Zhang H., Qin J., Lan X., Zeng W., Zhou J., Huang T.-E., Xiao W.-L., Wang Q.-Q., Sun S., Su W. (2022). Handelin extends lifespan and healthspan of Caenorhabditis elegans by reducing ROS generation and improving motor function. Biogerontology.

[42] Balaban R.S., Nemoto S., Finkel T. (2005). Mitochondria, oxidants, and aging. Cell.

[43] Newsholme P., Cruzat V.F., Keane K.N., Carlessi R., de Bittencourt P.I.H. (2016). Molecular mechanisms of ROS production and oxidative stress in diabetes. Biochem. J..

[44] Xu A., Zhang Z., Ko S.H., Fisher A.L., Liu Z., Chen L. (2019). Microtubule regulators act in the nervous system to modulate fat metabolism and longevity through DAF-16 in *C. elegans*. Aging Cell.

[45] Blackwell T.K., Steinbaugh M.J., Hourihan J.M., Ewald C.Y., Isik M. (2015). SKN-1/Nrf, stress responses, and aging in *Caenorhabditis elegans*. Free Radic. Biol. Med..

[46] Wan Q.-L., Zheng S.-Q., Wu G.-S., Luo H.-R. (2013). Aspirin extends the lifespan of *Caenorhabditis elegans* via AMPK and DAF-16/FOXO in dietary restriction pathway. Exp. Gerontol..

[47] Wang S., Yi X., Wu Z., Guo S., Dai W., Wang H., Shi Q., Zeng K., Guo W., Li C. (2022). CAMKK2 defines ferroptosis sensitivity of melanoma cells by regulating AMPK—NRF2 pathway. J. Investig. Dermatol..

[48] Koyama T., Nakajima C., Nighimoto S., Takami M., Woo J.-T., Yazawa K. (2012). Suppressive effects of the leaf of *Terminalia catappa* L. on osteoclast differentiation in vitro and bone weight loss in vivo. J. Nutr. Sci. Vitaminol..

[49] Jang M., Choi S.I. (2022). Schisandrin C isolated from *Schisandra chinensis* fruits inhibits lipid accumulation by regulating adipogenesis and lipolysis through AMPK signaling in 3T3-L1 adipocytes. J. Food Biochem..

[50] Wang K., Chen S., Zhang C., Huang J., Wu J., Zhou H., Jin L., Qian X., Jin J., Lyu J. (2018). Enhanced ROS production leads to excessive fat accumulation through DAF-16 in *Caenorhabditis elegans*. Exp. Gerontol..

[51] Bai J., Farias-Pereira R., Zhang Y., Jang M., Park Y., Kim K.H. (2020). *C. elegans* ACAT regulates lipolysis and its related lifespan in fasting through modulation of the genes in lipolysis and insulin/IGF-1 signaling. BioFactors.

