# Supplementary material for: Indian Almond (Terminalia catappa Linn.) Leaf Extract Extends Lifespan by Improving Lipid Metabolism and Antioxidant Activity Dependent on AMPK Signaling Pathway in Caenorhabditis elegans under High-Glucose-Diet Conditions
Source: Antioxidants (Basel). 2023 Dec 20;13(1):14. doi: 10.3390/antiox13010014 (PMC10812731; doi:10.3390/antiox13010014)
Supplement: Supplementary file 1 [file antioxidants-13-00014-s001.zip › antioxidants-2707248-supplementary.pdf]

Indian almond (*Terminalia catappa* Linn.) leaves extract healthspan by improving lipid metabolism and antioxidant activity dependent on AMPK signaling pathway in *Caenorhabditis elegans* under high-glucose diet conditions

Yebin Kim[1], Seul-bi Lee[1], Myogyeong Cho[1], Soojin Choe[2] and Miran Jang[1, 2*]

1 Department of smart food and drug, Inje University, Gimhae 50834, Republic of Korea
2 Department of Food Technology and Nutrition, Inje University, Gimhae 50834, Republic of Korea
* Correspondence: mrjangl@inje.ac.kr; Tel.: +82-55-320-3234

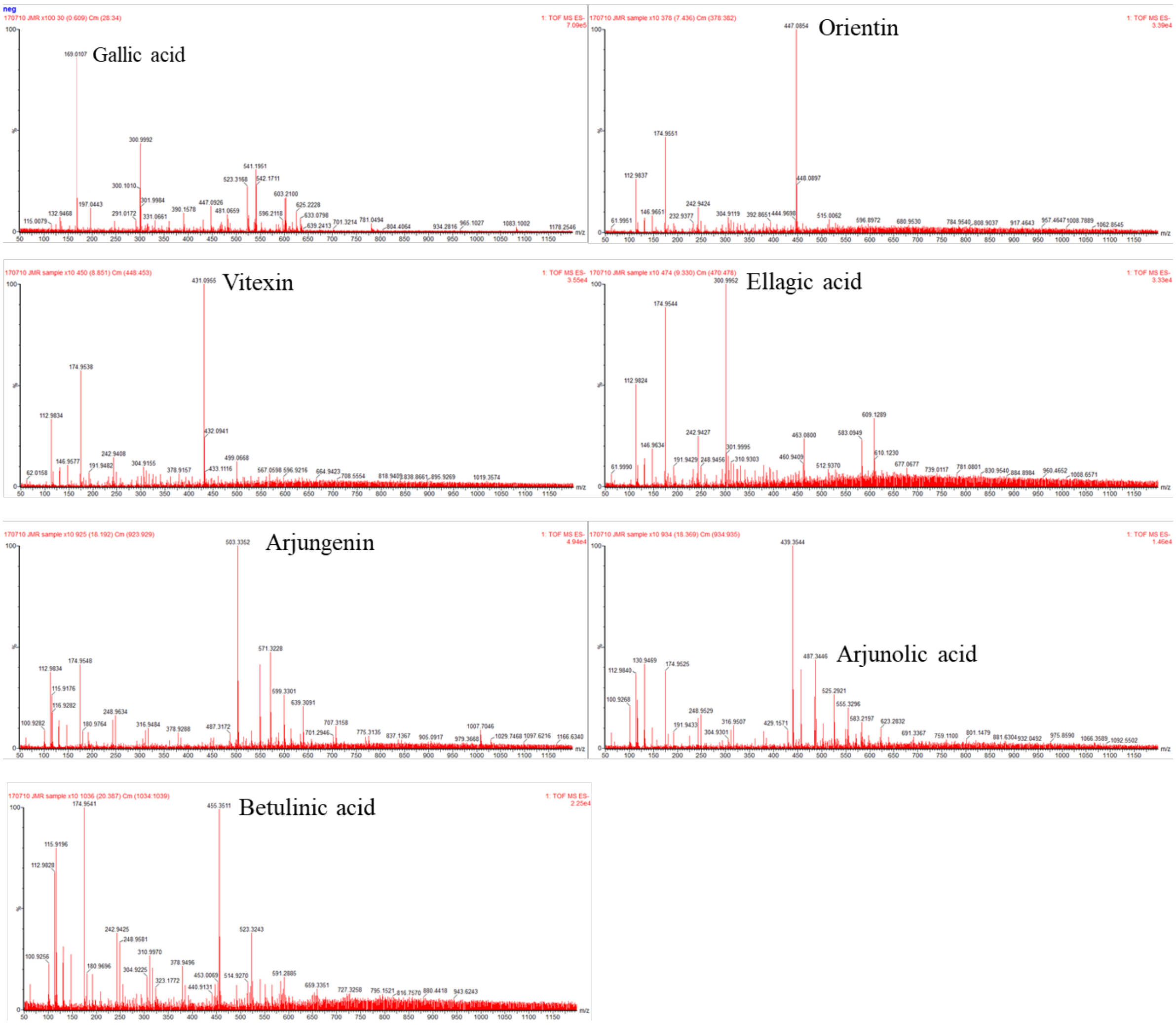

Figure S1. UPLC-QTOF-MS fragments of TCE.

Table S1. Influence of TCE on survival rate in various strains

Genotypes	TCE concentrations (μg/mL)	Conditions	Mean lifespans[a]	Maximum lifespans[b]	P-value[c]
N_2	0	Normal	18.13 ± 1.22[e]		
	6.25		23.94 ± 1.81		< 0.05
	12.5		26.80 ± 1.95		< 0.001
	25		22.04 ± 1.54		0.0520
	0	2% Glucose	12.73 ± 0.46		
	6.25		18.83 ± 0.85		< 0.001
	12.5		17.40 ± 0.81		< 0.001
	25		19.32 ± 0.69		< 0.001
daf-16	0	Normal	15.64 ± 0.45		
	6.25		17.47 ± 0.52		< 0.05
	12.5		18.29 ± 0.84		< 0.05
	25		18.52 ± 1.18		< 0.05
	0	2% Glucose	17.46 ± 0.45		
	6.25		18.61 ± 0.63		< 0.05
	12.5		16.02 ± 0.49		0.1207
	25		18.43 ± 0.60		< 0.05
skn-1	0	Normal	12.82 ± 0.41		
	6.25		12.88 ± 0.38		0.9790
	12.5		13.26 ± 0.35		0.7669
	25		11.61 ± 0.44		< 0.05
	0	2% Glucose	11.04 ± 0.27		
	6.25		11.21 ± 0.25		0.6049
	12.5		11.14 ± 0.33		< 0.05
	25		12.36 ± 0.30		< 0.001
aak-1	0	Normal	16.04 ± 1.20		
	6.25		21.18 ± 1.04		< 0.001
	12.5		19.22 ± 1.09		0.1534
	25		17.82 ± 1.11		0.4360
	0	2% Glucose	15.68 ± 1.19		
	6.25		15.94 ± 1.19		0.9537
	12.5		16.65 ± 1.50		0.9844
	25		14.98 ± 1.63		0.6797
atgl-1	0	Normal	26.80 ± 0.86		
	6.25		28.63 ± 1.06		< 0.05
	12.5		29.35 ± 1.04		< 0.01
	25		28.13 ± 1.24		0.2019
	0	2% Glucose	25.09 ± 1.03		
	6.25		25.58 ± 1.08		0.9037
	12.5		24.77 ± 1.28		0.7051
	25		24.49 ± 1.35		0.4260

Worms were treated with TCE from the L4 stage. Survival was recorded every other day until all worms died. The statistically data was generated by the OASIS application ($p < .05$).

a: the mean lifespan was referred to the time when the survival rate dropped to 50%.

b: the maximum lifespan means end points (days) of the lifespan of the worm.

c: P-values were analyzed by log-rank (Mantel-Cox method) tests.

d: days ± standard error
